# Leaf oil under stress: divergent lipid synthesis and stabilization strategies in tobacco

**DOI:** 10.1093/plphys/kiaf200

**Published:** 2025-07-21

**Authors:** Sara Shakir

**Affiliations:** Assistant Features Editor, Plant Physiology, American Society of Plant Biologists; UMR Biologie du Fruit et Pathologie, INRAE, Université de Bordeaux, Villenave d’Ornon 33882, France

Plant-derived oils are in increasing global demand for food, animal feed, and renewable biofuels thanks to their nutritional value and production scalability (IEA 2021). In traditional crops, neutral lipids such as triacylglycerols (TAGs) and sterol esters are primarily stored in seeds in cytoplasmic lipid droplets (LDs) (Xu et al. 2020). These neutral lipids are readily available fatty acid reservoirs that are crucial for plant growth and development. Cytoplasmic LDs are spherical organelles composed of a neutral lipid core enclosed in a monophospholipid layer coated with integral and non-integral proteins, such as oleosins in seeds and LD-associated proteins (LDAPs) in vegetative tissues. These proteins stabilize LD size and neutral lipid homeostasis (Pyc et al. 2017).

TAGs are energy-dense molecules composed of 3 fatty acids esterified to a glycerol. Their biochemical properties—notably, being highly reduced hydrocarbons—make them particularly attractive for biofuel production, with nearly twice the energy content per gram compared to carbohydrates (Santner et al. 2023). However, less than 1% of the total TAG is stored in vegetative tissues in most plants. Because vegetative tissues make up the majority of plant biomass and can be produced more rapidly and continuously than seeds, engineering oil accumulation in leaves and stems offers a far greater potential for scalable biofuel production. There is high interest in understanding and re-engineering lipid metabolism through strategies known as push, pull, package, and protect (Vanhercke et al. 2019). These strategies include boosting glycolysis and de novo fatty acid biosynthesis in plastids through overexpression of key transcription factors such as WRINKLED1 (WRI1) (push), redirecting existing lipid intermediates into TAG assembly at endoplasmic reticulum via catalytic activity of acyltransferases (pull), storage of neutral lipids in stable cytoplasmic LDs (package), and minimizing TAG turnover in peroxisomes by downregulating lipases (protect).

These metabolic re-engineering strategies successfully mimic oilseed metabolic fluxes and redirect carbon toward increased TAG accumulation in leaves up to 30%, enabling the development of highly valuable plants with high leaf oil (HLO), including tobacco, sorghum, and sugarcane (Vanhercke et al. 2014, 2019; Zale et al. 2016). However, evaluation of their performance as oil biofactories in agricultural settings is lacking. Abiotic stresses, such as drought and heat, alter primary metabolism and lipid homeostasis in plants (Du et al. 2020). For instance, drought affects stomatal conductance and carbon assimilation and triggers senescence, processes intimately tied to carbon allocation and lipid remodeling. Stress-responsive genes such *LDAPs* are known to modulate neutral lipid biosynthesis and storage during drought (Kim et al. 2016). These lipid biosynthesis and storage processes are believed to sequester free fatty acids generated during membrane remodeling and protect cells from oxidative damage, especially under drought.

Recently, in *Plant Physiology*, Zhang et al. (2025) combined molecular and physiological approaches to investigate how lipid-inducing transgenes modulate endogenous lipid metabolism in HLO tobacco and how these modifications intersect with plant responses to drought (Fig. 1). First, the authors assessed the physiological responses of wild-type (WT) and HLO tobacco plants under well-watered (control) and sustained water deficit (drought) conditions. Interestingly, HLO plants consistently maintained lower and more stable stomatal conductance than WT, indicating tighter control over water loss. Notably, HLO plants were smaller in stature under controlled conditions, a growth trade-off that appeared beneficial under drought. Their reduced leaf area lowered canopy transpiration during water stress, helping conserve soil moisture and resulting in a smaller biomass penalty than WT. Indeed, water stress turned out to be more than just a limiting factor. Remarkably, both genotypes accumulated more TAG under water stress: HLO plants exhibited up to 61% higher TAG in older leaves, while WT plants doubled TAG levels in younger leaves, pointing to distinct strategies for lipid storage in response to stress. These molecular changes were tightly linked to carbon partitioning, a process where carbon fixed by photosynthesis is allocated either as sucrose in cytoplasm or starch in chloroplast. Under drought, both genotypes showed reduced starch accumulation, but only HLO plants maintained total carbon and significantly increased soluble sugars, suggesting a shift in resource allocation; starch breakdown funnels carbon into soluble sugar pools, which in HLO may then feed lipid biosynthesis (Du et al. 2020). In WT plants, however, reduction in total carbon and starch is likely due to decreased photosynthetic capacity following stomatal closure.

**Figure 1.**
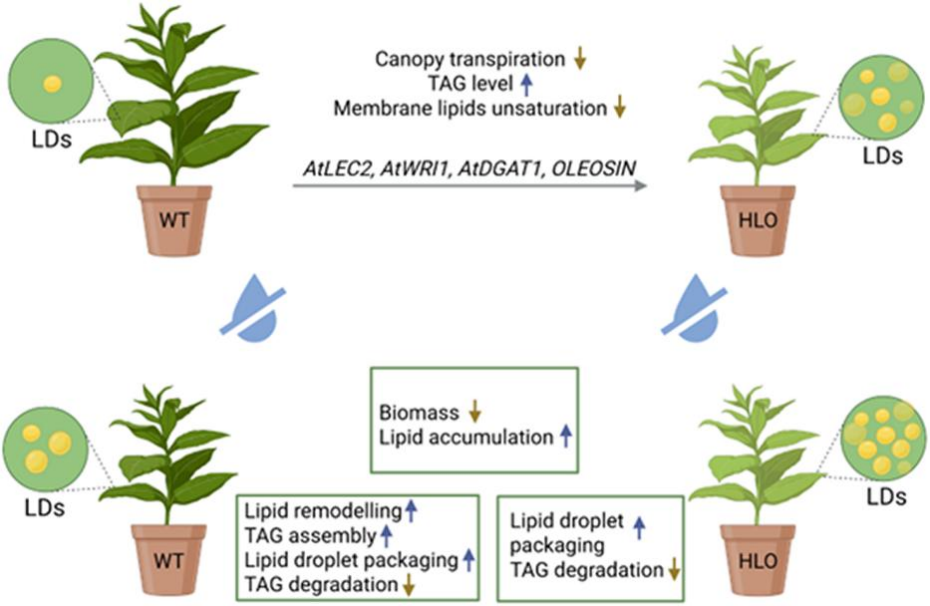
Distinct TAG accumulation strategies in WT and HLO tobacco under water stress. Under water stress conditions, WT and HLO tobacco plants employ divergent lipid metabolic strategies. WT plants show enhanced lipid remodeling, increased TAG assembly, and reduced degradation. In contrast, HLO plants primarily stabilize pre-accumulated TAGs by increasing the expression of LD packaging proteins (e.g. oleosin) and suppressing TAG breakdown. Both genotypes exhibit increased lipid accumulation and biomass reduction under drought, with HLO plants further benefiting from reduced canopy transpiration and stable membrane lipid fluidity. Gene constructs (*AtLEC2*, *AtWRI1*, *AtDGAT1*, *OLEOSIN*) drive constitutive TAG production in HLO plants. A larger number of LDs in HLO leaves reflects enhanced storage under both control and stress conditions. Water drop symbol with a slash represents drought. Figure modified from Zhang et al. 2025.

Building on this metabolic shift, Zhang et al. (2025) used liquid chromatography-mass spectrometry and gas chromatography-flame ionization detector–based lipidomic profiling to show that drought reprogrammed fatty acid composition and membrane lipid dynamics in both genotypes. In WT plants, drought induced a sharp decline in trienoic fatty acids such as α-linolenic acid and α-linolenic acid–containing polar lipids. Trienoic fatty acids are major polyunsaturated fatty acid species in the membrane lipids in plant cells that represent an adaptive strategy to abiotic stresses by maintaining optimal membrane fluidity and integrity (Murakami et al. 2000). In contrast, HLO plants maintained membrane polar lipid stability and accumulated more saturated and monounsaturated fatty acids in both control and drought conditions, suggesting their intrinsic capacity to maintain optimum membrane fluidity.

Transcriptomic analysis revealed a broad reprogramming of lipid metabolism through distinct molecular mechanisms between the genotypes. In WT plants, genes and transcription factors involved in TAG biosynthesis (e.g. *WRI1*, *diacylglycerol acyltransferase2/DGAT2*) and lipid droplet packaging (*LDAP1*) were activated during drought. The results suggest a remodeling-based response in which carbon and membrane lipids are redirected toward de novo TAG synthesis during stress. In contrast, the induction of lipid biosynthetic genes in HLO plants was minimal, likely because the system had already been primed by the transgene overexpression. Instead, genes encoding LD-stabilizing proteins such as oleosin1 and oleosin5 were upregulated in HLO plants. Moreover, expression of TAG-degrading genes encoding lipases was downregulated in HLO plants, contributing to reduced TAG turnover and indicating the “protect, not produce” strategy. This strategic divergence highlights the flexibility of the plant lipid network and offers multiple entry points for future metabolic engineering.

Combined with previous knowledge on adaptive lipid metabolism under drought (Kim et al. 2016), these data on de novo synthesis and storage of neutral lipids contribute to our understanding of lipid mobilization and carbon partitioning in WT and HLO tobacco plants as part of an efficient drought response. In summary, TAG accumulation in leaves can be both a drought-responsive and drought-resilient trait, acting on distinct strategies in WT and HLO plants. WT plants rely on a dynamic remodeling process to produce more lipids in cells, whereas HLO plants have adapted a lipid protection strategy to cope with drought. The divergence highlights a key advantage of engineered systems: the ability to sustain oil accumulation without compromising physiological integrity under drought. HLO plants also conserve water and accumulate biomass relatively effectively, making them promising candidates for biofuel production in water-limited environments. Key challenges ahead include optimizing the balance between oil yield and plant growth and assessing HLO performance under combined field stresses such as drought, heat, and nutrient limitation. This also raises economically important questions such as whether stress-responsive TAG stabilization can be fine-tuned and transferred to other major food and oil crops. Ultimately, turning leaves into oil biofactories may no longer remain just an engineering feat; it is emerging as a smart survival strategy in a drier world.

## Data Availability

The data underlying this article are available in the article.
